# Microdissection of the A_h_01 chromosome in upland cotton and microcloning of resistance gene anologs from the single chromosome

**DOI:** 10.1186/s41065-017-0035-3

**Published:** 2017-05-18

**Authors:** Xinchuan Cao, Yuling Liu, Zhen Liu, Fang Liu, Yalei Wu, Zhongli Zhou, Xiaoyan Cai, Xingxing Wang, Zhenmei Zhang, Yuhong Wang, Zhimin Luo, Renhai Peng, Kunbo Wang

**Affiliations:** 1 Tarium Universty, Alar, Xinjiang 843300 China; 2 State Key Laboratory of Cotton Biology/Institute of Cotton Research of Chinese Academy of Agricultural Sciences, Anyang, Henan 455000 China; 3 Anyang Institute of Technology, Anyang, Henan 455000 China; 4 College of Life Science, Zhengzhou University, Zhengzhou, Henan 450001 China

**Keywords:** Upland cotton, Chromosome microdissection, Microcloning, RGA

## Abstract

**Background:**

Chromosome microdissection is one of the most important techniques in molecular cytogenetic research. Cotton (*Gossypium* Linnaeus, 1753) is the main natural fiber crop in the world. The resistance gene analog (RGA) cloning after its single chromosome microdissection can greatly promote cotton genome research and breeding.

**Results:**

Using the linker adaptor PCR (LA-PCR) with the primers of rice disease-resistance homologues, three nucleotide sequences PS016 (KU051681), PS054 (KU051682), and PS157 (KU051680) were obtained from the chromosome A_h_01 of upland cotton (cv. TM-1). The Blast results showed that the three sequences are the nucleotide binding site-leucine rich repeat (NBS-LRR) type RGAs. Clustering results indicated that they are homologous to these published RGAs. Thus, the three RGAs can definitely be confirmed as NBS-LRR class of RGAs in upland cotton.

**Conclusions:**

Using single chromosome microdissection technique, DNA libraries containing cotton RGAs were obtained. This technique can promote cotton gene cloning, marker development and even the improvement of cotton genome research and breeding.

**Electronic supplementary material:**

The online version of this article (doi:10.1186/s41065-017-0035-3) contains supplementary material, which is available to authorized users.

## Background

Chromosome microdissection is one of the most important techniques in molecular cytogenetic research. Specific chromosome or chromosomal sections are isolated using a glass needle or laser under a microscope, and then are enzymatically digested and amplified to construct DNA library of a single chromosome or chromosomal section. Research focusing on a single chromosome or a chromosomal subsection can greatly reduces subsequent work, such as identifying, screening and minimizing the whole genome screening. This technique has been widely used in *Drosophila*, humans and many other animals since its establishment [1–8] Subsequently, the technique has been widely adapted to apply in herbaceous plants including barley, wheat, rice, and tomato [9–18] and woody plants such as pomelo and poplar [19, 20].

Plants have developed defensive mechanisms to protect themselves from pathogen infection through a number of evolutionary processes. The gene-for-gene hypothesis proposed by Flor is based on the interactions between pathogenic fungi and host plants and constitutes the theoretical basis of cloning avirulence genes from pathogens and resistance genes (R genes) from plants [21]. So far, many R genes have been cloned from different host plants using positional cloning and transposon tagging methods. However, considering the large number of physiological races of pathogens, transposon tagging and positional cloning methods are clearly inefficient. Thus new strategies and methods should be adopted to accelerate the cloning of disease R genes. Due to the conserved domains of R genes, homologous sequence amplification or the homologous sequence-based candidate gene approach would be a good choice; actually, these techniques have been quickly adopted by the scientific community. A great progress has been made in recent years for obtaining disease RGAs from many plant species [22–28]. Additionally, some of these RGAs were used as probes for linkage analysis and positioning [22–24, 27].

As the primary natural fiber crop, cotton (*Gossypium hirsutum*) plays an important role in the world’s economy. However, cotton cells contain large amounts of secondary metabolites, and their chromosomes are small in size and nearly identical to each other. These prevent in somehow to well prepare the chromosomes from the cells and clearly distinguish them from their karyotypes, and thus cytogenetic research of cotton is still lagging behind other plant species, such as rice and wheat. As a typical tetraploid plant species [29–35], there are two sub-genome (A_1_A_1_D_1_D_1_, 2n = 4× = 52) and high number of nucleotide sequence repeats in cotton genome. There are greater uncertainties in interpreting whole genome while assembling or annotating [33, 34]. Microdissection of a single chromosome or its subsections using direct micromanipulation techniques and gene microcloning through molecular biology should be one easy way to slove this problem. However, currently, there is only one report about single chromosome microdissection that was from somatic cells [36], there is no any report on chromosome microdissection from pollen mother cells (PMC) and on gene microcloning from single chromosome.

There are many important genes are closely related to disease resistance, fiber development, fiber quality and yield in the A_h_01 chromosome of TM-1 upland cotton [37–39]. In this study, the A_h_01chromosome was microdissected from the A_h_01 monosome materials derived from TM-1 (a genetically standard line of upland cotton) using the laser method. A DNA pool was constructed from the single chromosome by amplifying DNA using linker adaptor polymerase chain reaction (LA-PCR). RGAs from this chromosome were then cloned.

## Methods

### Plant materials

A accession of A_h_01 monosome, developed from the genetically standard line of upland cotton at Texas A&M University (U.S.) [40], was used as the primary plant materials. The accession was grown in the Greenhouses at the Institute of Cotton Research, Chinese Academy of Agricultural Sciences, (ICR-CAAS) (Anyang, Henan, China) and is also maintained in the National Wild Cotton Nursery located at Sanya City, Hainan Island, China.

### SSR markers and primers

The chromosome-A_h_01-specific BAC clone 52D06 was provided by Professor Tianzhen Zhang of Nanjing Agricultural University. The primers of corresponding simple sequence repeat (SSR) marker BNL3580 (F primer: CTTGTTTACATTCCCTTCTTTATACC; R primer: CAAAGGCGAACTCTTCCAAA), degenerate specific primers P_1_ (5′-GATCCTGAGCTCGAATTCGACCC-3′) and P_2_ (5′-GGGTCGAATTCGAGCTCAG-3′) were synthesized by Shanghai Sangon Biotech Inc. [41].

### Preparation of mitotic metaphase chromosomes

Mitotic metaphase chromosomes were prepared according to a previous report [36] with a few modifications. The slides prepared were kept at −20 °C for a long-term storage or at 4 °C for a short period storage. Slides were baked at 60 °C overnight immediately before use.

### Preparation of film-slides

Sampling, fixing, and enzymatic hydrolysis of flower buds were performed according to the protocol of Peng et al. [36]. Enzymatically digested anthers were smeared on film-slides as previously described [36].

### Microdissection of single chromosome and LA-PCR amplification

Single chromosome was microdissected using the CellCutPlus Laser micromanipulation system (MMI Company, Swiss) and LA-PCR amplification was conducted as previously described [36]. Positive (~10 pg of genomic DNA added to the initial template) and negative controls (no genomic DNA added to the initial template) were also set up.

### Agarose electrophoresis

Two rounds of LA-PCR products were separated through electrophoresis with 1% agarose at 100 V for 30 min. LA-PCR products were observed and photographed under UV light after 40 min staining with ethidium bromide.

### Southern hybridization

Southern hybridization was conducted with PCR products, partially digested genomic DNA, positive control (PCR product from genomic DNA as template) and negative control (no template PCR reaction) [42].

### SSR amplification

The amplified pool of A_h_01 chromosomes and second LA-PCR products were amplified using the chromosome- A_h_01- specific SSR primer respectively. The amplified products were checked by polyacrylamide gel electrophoresis (PAGE).

### Fluorescence in situ hybridization

Dual-color FISH (fluorescence in situ hybridization) and the detection of metaphase chromosome specimens were performed according to a previous [36]. The second round of LA-PCR products labeled with DIG (Digoxigenin-11-dUTP, Roche) and specific Biotin (Biotin-16-dUTP) labeled bacterial artificial chromosome (BAC) clones (52D06) were used as probes, which were detected by Anti-Digoxigenin-Rhodamine (red) and FITC-Anti-Biotin (green) (Roche Diagnostics, USA), respectively. Cot-1 DNA was used to pre-hybridize for blocking the repetitive sequences. Chromosomes were counterstained by 4′, 6-diamidino-2-phenylindole (DAPI) in VECTASHIELD anti-fade solution (Vector Laboratories, Burlingame, CA). The hybridization signals were observed using a fluorescence microscope with a change-coupled device (CCD) camera (Zeiss Axiokop2 plus). The images were adjusted using Adobe Photoshop CS3 software.

### Cloning and analysis of RGAs

RGA sequences were obtained by PCR with the A_h_01 chromosome second round LA-PCR product as template and P_1_ and P_2_ as degenerate specific primers. Positive control (about 10 pg of genomic DNA was added to the initial substrate) and negative control (no template) reactions were also performed. The PCR products were examined by agarose gel electrophoresis and Southern hybridization. The target bands of the PCR products were recovered. Positive clones were obtained, and sequenced, and the sequences were used as a probe BLAST search of homologues in NCBI Genbank database.. Screened homologous RGA clones were sequenced by Shanghai Sangon Biotech Inc. Introns were annotated with ORFinder. The sequences were queried against the tetraploid *Gossypium hirsutum* genome sequcence [33, 34]. The final obtained sequences were submitted to Genbank. BlastN search was performed in GeneBank using these sequences, and a sequence cluster was created by Phylip.

## Results

### Chromosomes preparation and microdissection

**Fig. 1:**
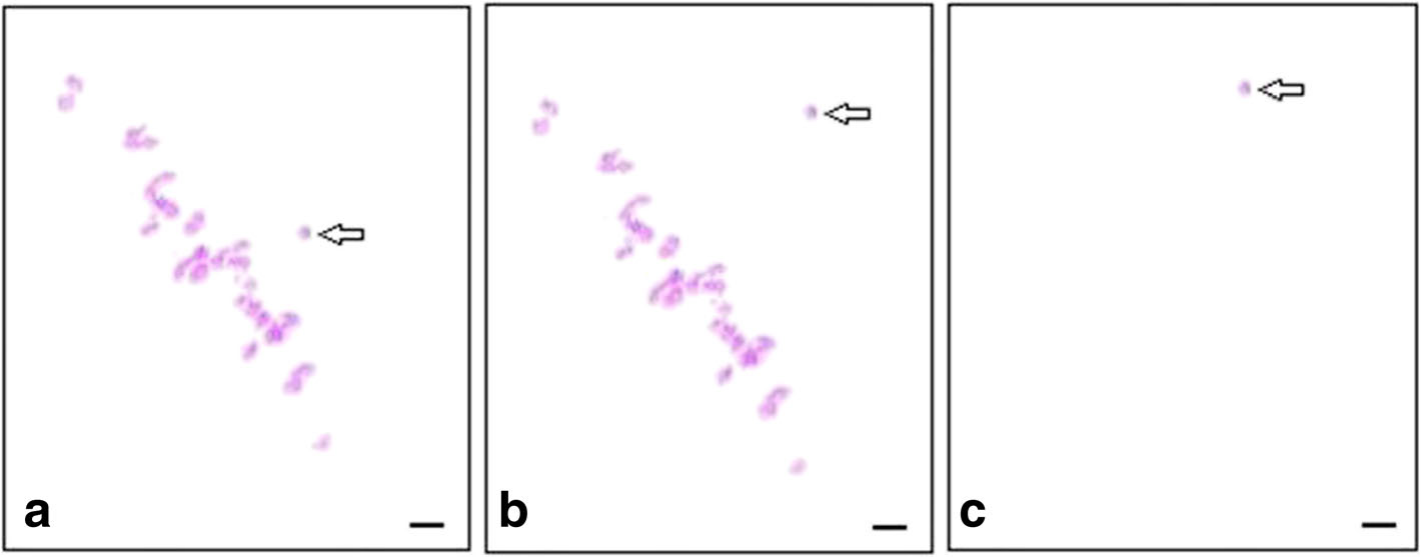
Microdissection and collection of single mono-chromosomes by CellCut Plus laser manipulation. **a** Film-slide preparations of meiotic metaphase I chromosomes with one monomer chromosome (A_h_01). **b** Film-slide preparations of meiotic metaphase I chromosomes with one microdissected chromosome. **c** The microdissected chromosome on the cap of a collection tube. *Arrow* indicates the A_h_01 chromosome. *Bar*: 5 μm

PMCs moderately digested in an enzymatic mixture were stained with carbolfuchsin. The PMCs at metaphase I were used for chromosome preparation (Fig. 1a). The target chromosome A_h_01was initially found under low magnification, and then captured under high magnification for collection in a tube containing10 μL proteinase K (50 ng·μL^−1^) solution. The protocols for cutting and collecting chromosomes are shown in the Figure 1. For comparison, other chromosomes in metaphase I were simultaneously collected in different tubes for SSR-amplified proof after second LA-PCR amplification.

### LA-PCR amplification of chromosomal DNA

**Fig. 2:**
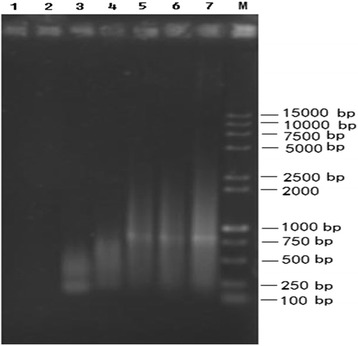
Agarose gel electrophoresis of LA-PCR products. **1**, **2:** Negative controls **3** Product from the first round LA-PCR. **4**, **7:** Positive controls. **5**, **6:** Products from the second round LA-PCR. **M:** DNA marker

Two rounds of LA-PCR were conducted to amplify the A_h_01 chromosomal DNA. Electrophoresis results (Fig. 2) revealed that a weak DNA smear with sizes ranging from 200 to 1000 bp after the initial LA-PCR (Fig. 2, lane 3), and a strong DNA smearing pattern with sizes ranging from 300 to 2500 bp were generated after the second LA-PCR (Fig. 2, lane 5, 6), because of more products. For the negative controls, there were no bands (Fig. 2, lane 1, 2). The positive control produced a weak initial band (Figure 2, lane 4) and an obvious smearing pattern after the second LA-PCR (lane 7 in Figure 2). These results indicated that the A_h_01 chromosome was amplified successfully.

### Southern blot analysis

**Fig. 3:**
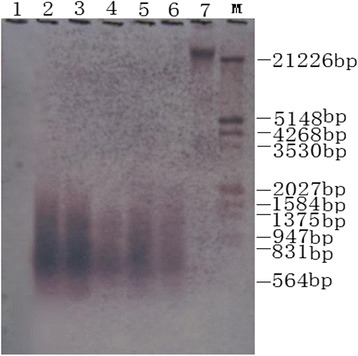
Southern blotting of products from the second round LA-PCR. **1:** The negative control. **2**, **3:** Positive controls. **4**, **5**, **6:** The second round LA-PCR products. **7:** *Eco*RI digested genomic DNA. **M:** DNA marker

Enzyme-labeled upland cotton genome was used as a probe, and the second LA-PCR products were verified by Southern hybridization with negative and positive controls (Fig. 3). Results of Southern blot showed that the second products of LA-PCR (Fig. 3, lane 4–6) and positive control (Fig. 3, lane 2, 3) had obvious bands, indicating that the amplification products from *G. hirsutum* genome were ranging from 300 to 2000 bp; that was consistent with the results from agarose gel electrophoresis. There were no bands in the negative control PCR (Figure 3, lane 1).

### Verification of SSR amplification

**Fig. 4:**
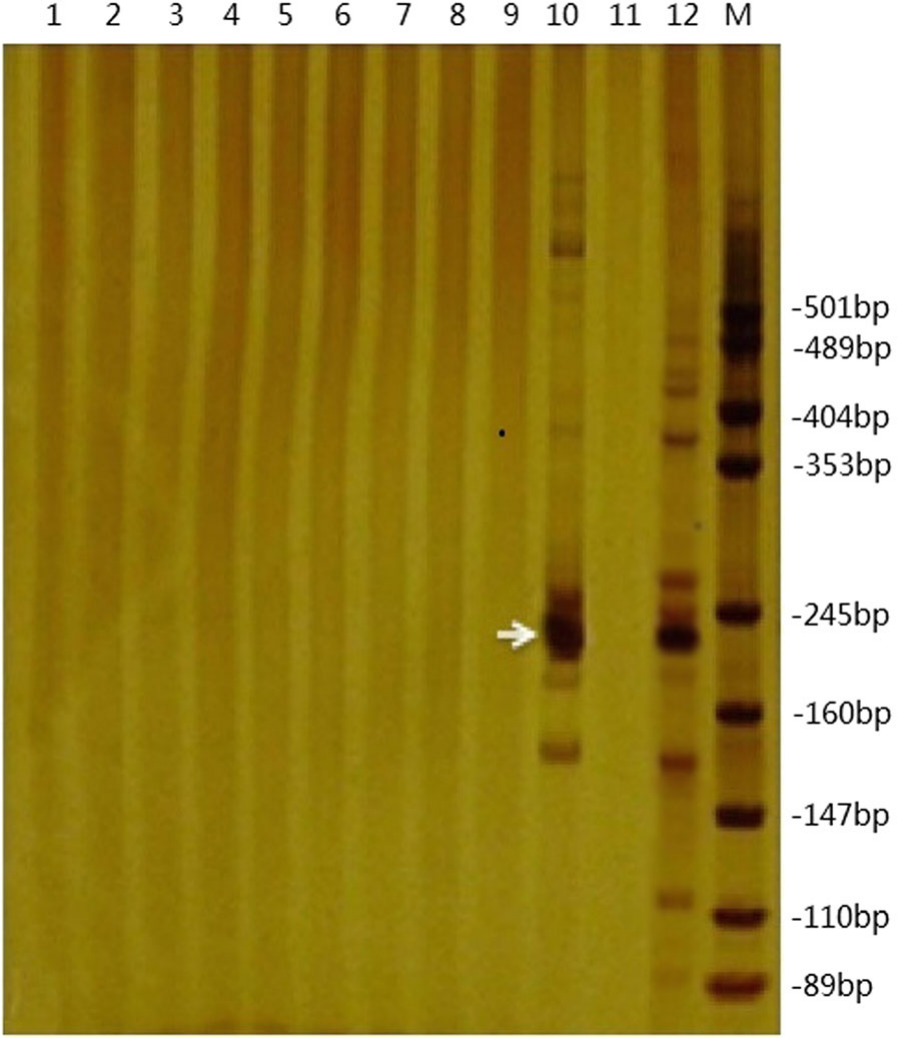
PAGE of SSR primer amplification product from single chromosome pool. **1**–**9:** SSR primer amplification products from partial other chromosomes pool with A_h_01 chromosome specific primer. **10:** SSR primer amplification products from single chromosome pool with chromosome A_h_01 special primer (arrow indicated). **11:** The negative control. **12:** The positive control. **M:** DNA marker

Specific SSR primer from A_h_01 chromosome was selected to amplify the second LA-PCR products of chromosome A_h_01 and some other chromosomes. Results were checked by PAGE, and it was observed that the A_h_01chromosome could amplify a specific band (240 bp), as shown in Fig. 4 (Fig. 4, lane 10). The similar band was obtained using the genome DNA as positive control (Lane 12), no band in negative control (Fig. 4, lane 11) and partly others chromosomes (Fig. 4, lane 1–9).

### Fluorescence in situ hybridization

**Fig. 5:**
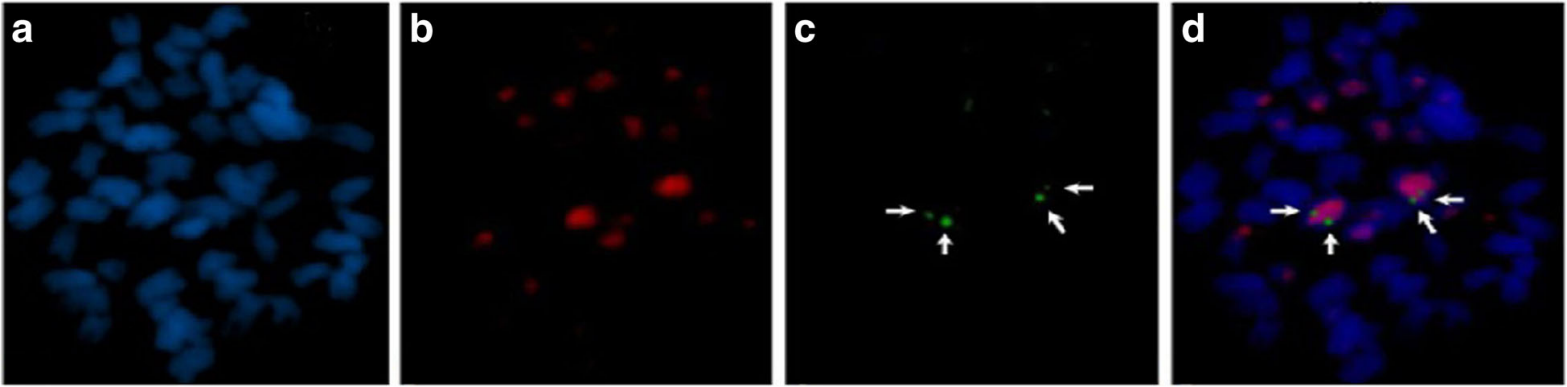
FISH signals of products from the second round LA-PCR. **a** Chromosomes stained with DAPI. **b** Signals fromproducts of LA-PCR II (*red*). **c** Signals fromchromosome A_h_01 specific BAC (*green, arrow* indicated). **d** Signals from dual-FISH

Dual-color FISH was performed using DIG-labeled products of LA-PCR II and specific Biotin-labeled A_h_01 chromosome BAC clone (52D06) to probe the metaphase chromosome slide. As shown in Fig. 5, the target chromosomes were accurately identified by means of the specific BAC clone as well as products of LA-PCR II. Meanwhile, partial other chromosomes had weak signal (red light), indicating homologous sequence on these chromosomes.

### Isolation of RGAs

**Fig. 6:**
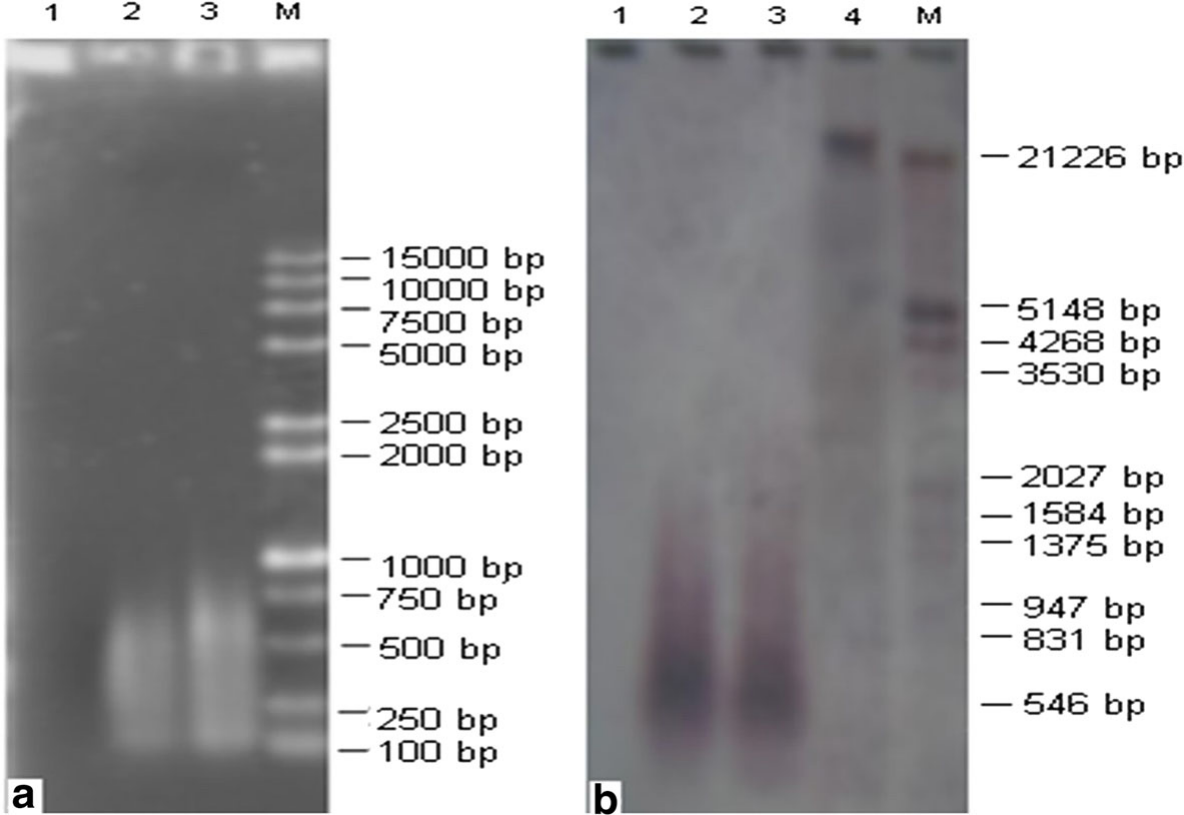
Agarose gel electrophoresis (**a**) and Southern blotting (**b**) of P1/P2 primer PCR products. **A**-**1**, **B-1**: Negative controls. **A**-**2**, **B**-**2:** Single A_h_01 chromosome as DNA template. **A**-**3**, **B**-**3**: Positive controls using 10 pg *G. hirsutum* genomic DNA as template. **B**-**4:** *Eco*RI digested genomic DNA of *G. hirsutum*. **M:** DNA marker

RGAs were isolated using PCR with P_1_ and P_2_ as primers, and TM-1 upland cotton genomic DNA and the second round LA-PCR A_h_01 chromosome pool as templates, respectively. Products were detected by gel electrophoresis and Southern hybridization. Using genomic DNA as positive control, a slightly wider DNA smear with sizes ranging from 400 to 1000 bp and a major band of 550 ~ 700 bp was generated (Fig. 6a, Lane3). A narrow DNA smear with sizes ranging from 550 to 800 bp and a main band of 650 bp was generated using the second LA-PCR products as template (Fig. 6a, Lane 2). Southern blot results demonstrated that the products come from the genome of upland cotton (Fig. 6b).

### Cloning and validation of RGAs

**Fig. 7:**
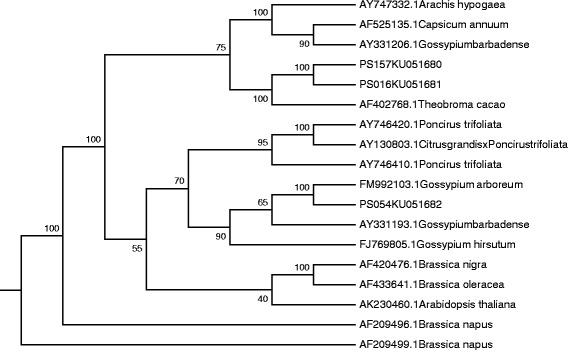
Cluster analysis of single chromosome RGA nucleotide sequenceswith those from other species

Main bands of the PCR products were recovered and cloned. Two hundred positive clones (PS001 ~ PS200) were obtained and sequenced, followed by BLAST analysis. Three sequences [(Additional file 1) named PS016 (Genbank ID: KU051681), PS054 (Genbank ID: KU051682) and PS157 (Genbank ID: KU051680)] contained a conserved domain common to the NBS-LRR R genes in plant. Clustering results showed that they were homologous to these published RGAs (Fig. 7). Alignment was made with others RGAs from NCBI (Additional file 2), the results also definitely confirmed that the three RGAs were the NBS-LRR class of RGAs in cotton.

## Discussion

### Identification and microdissection of a single chromosome

Accurate identification of the target chromosome is a key step in chromosome microdissection and cloning. Identification of the target chromosome has mainly relied on the morphological features such as monosome, trisome, nullisome and shape-specific chromosomes [9–11, 43–47]. Chromosome banding technique has also been reported as a method to identify chromosomes [48], but this method has not widely used in plants. In this study, monosome chromosome in meiotic metaphase I were easy to identify and isolate from other chromosomes.

There are three approaches reported for chromosome isolation. One is flow cytometry, which has facilitated the dissection of large genome into smaller and defined segments for the purpose of gene discovery and genome sequencing in plants [13]. Nevertheless, this method not only requires expensive instrumentation, but also fails to distinguish chromosomes with similar morphological characteristics from one to another, which limit its application in plants to some extent. The second approach is the glass needle method, which involves in dissection of the target chromosome under an optical microscope by a glass needle. The approach is easily operate and independent from high-end instrumentation, which has resulted in effective and widespread application in plants [16, 20]. However, the approach requires the operator to be trained well enough, or there should be much deviation operated by different persons or even in different personal statuses by an operator. The third method is laser cutting [44, 49], in which chromosome specimens are dispersed onto a special carrier covered with a membrane for dissection and collection. In most cases, dissection is much easier than collection. In this study, the CellCutPlus Laser microdissection system was applied to isolate the target chromosome. Initially, cotton chromosomes were spread on the microscope slide coated with film, and then a single chromosome was dissected and automatically collected in a microcentrifuge tube with a sticky cap. This method has a high efficiency and a low risk of contamination.

### Confirmation of the chromosomal DNA

LA-PCR is a powerful tool for the amplification of long DNA segments, and it has been widely used in molecular biology [18, 50, 51]. In this research, A_h_01 chromosomal DNA was acquired by LA-PCR after microdissection of the target chromosome. Prior to subsequent steps, the PCR products were examined by agarose electrophoresis, Southern blot analysis, SSR primer confirmation and confirmed by FISH. Combining several confirmation methods could achieve multiple analyses, and ensure that amplification products were from the target chromosome.

### Significance of generating RGAs from specific single chromosome of cotton

R genes have been isolated from the whole genome or its cDNA in woody plants [7, 19, 20, 39]. In this study, RGAs were isolated from a single chromosome dissected from upland cotton, clarifying the source and location. Efficiency of downstream work was greatly improved due to the isolation of a single chromosome from the entire genome. It has been reported that the R genes family frequently clusters on a certain chromosomal segment [52]. Acquired RGAs could be transformed to molecular marks, and serve to construct a genetic map due to the clear linkage relationship from one chromosome. In addition, it will contribute to the development of map-based cloning, thus, generating RGAs from a specific chromosome has many advantages.

## Conclusions

Although cotton is one major crop in the world like rice, wheat and maize, its cytogenetic studies falls much behind others due to the smaller and identical chromosomes in morphology as well as large amounts of secondary metabolites within its cells. All these factors make the cotton chromosome preparation difficult. Here, we successfully developed a technique to separate a single chromosome from upland cotton PMC (monosome cells) with the laser cutting. Using this technology, we also microcloned three RGAs from the DNA pool constructed with the single chromosomes (A_h_01). The three RGAs belong to the nucleotide binding site-leucine rich repeat (NBS-LRR) gene family. The techniques will promote the cloning of cotton R genes and marker assistant improvement of cotton genetics and breeding.

## Additional files

Additional file 1: The nucleotide sequences of three newly identified RGA genes. (TXT 1 kb)

Additional file 2: Alignment of the three RGAs. (PDF 143 kb)
