# Supplementary material for: Microdissection of the Ah01 chromosome in upland cotton and microcloning of resistance gene anologs from the single chromosome
Source: Hereditas. 2017 May 18;154:13. doi: 10.1186/s41065-017-0035-3 (PMC5437636; doi:10.1186/s41065-017-0035-3)
Supplement: Supplementary file 2 — Alignment of the three RGAs. (PDF 143 kb) [file 41065_2017_35_MOESM2_ESM.pdf]

```
                                          10        20        30        40        50        60        70        80
                                 ....|....|....|....|....|....|....|....|....|....|....|....|....|....|....|....|

AF209496.1|Brassica_napus        ------------GGTGGGGTTGGGAAGACAACCCTTTTCAAGAAAATCCACAATAGGTTTGCTGGAACAGCTGAAAAGTT
AF209499.1|Brassica_napus        -----------TGGTGGGGTTGGGAAAACAACCCTTTTCAAGAAAATCCACAATAGGTTTGCTGGAACAGCTGAAAAGCT
AF420476.1|Brassica_nigra        ------------GGGGGGGTGGGGAAGACGACACTGCTCACACAAATCAACAATAAATTTCTCAAGAAAAAGGATGCCTT
AF433641.1|Brassica_oleracea     ------------GGGGGGGTGGGGAAGACGACTCATCTCACACAAATCAACAATAAACTTTTCAAGAAAAAGAATGTTTT
AY746420.1|Citrus  trifoliate    ------------GGGGGAGTAGGGAAGACGACTCTGTTAACCAAAATCAACAACAAGCTTCTTGGTGCACCAAATGGTTT
AY130803.1|Citrus  maxima        ------------GGGGGGGTGGGGAAGACGACTCTGTTAACCAAAATCAACAACAAGCTTCTTGGTGCACCAAATGGTTT
AY746410.1|Citrus  trifoliate    ------------GGCGGGGTGGGGAAGACTACCCTACTAACGCAAATCAACAACAAATTCCTTGATGCGCCAAACGATTT
FM992103.1|Gossypium  arboreum   --------------------GGCAGA---CACACTCTTGACCAAACTCAACAACAAGTTCAGCACCACACCGAATGGTTT
PS054                            --------------------GGCAGAAATCACACTCTTGACCAAACTCAACAACAAGTTCAGCACCACACCGAATGGTTT
AY331193.1|Gossypium_barbadense  ---------------GGGGTGGGGAAGACAACTCTCTTGACCAAACTCAACAACAAGTTCAGCACCACACCGAATGATTT
FJ769805.1|Gossypium_hirsutum    ------------GGGGGGGTGGGGAAGACGACAATCTTGACCAAACTCAACAACAAGTTTAGCACCAAACCGAATAATTT
AK230460.1|Arabidopsis  thaliana CTTTATGGTATGGGGGGAATAGGAAAAACCACCCTCTTAGAAAGTCTCAACAACAAATTTGTTGAACTGGAGAGTGAATT
PS157                            ---AGCGGGATGGGGGGGGTGGGGAAGACTACCATTATGAAGGATGTCCATAATAGGTTGTTGGA---AGAGAGTAAATT
PS016                            GGGAAAGGGATGGGGGGGGTGGGGAAGACTACCATTATGAAGGATGTCCATAATAGGTTGTTGGA---AGAGAGTAAATT
AF402768.1|Theobroma_cacao       ------GGGATGGGGGGGGTGGGGAAAACAACCATAATGAAGCATGTCCATAATAGGTTGTTGAA---AGAAGATAAGTT
AY747332.1|Arachis  hypogaea     -----TGGGATGGGGGGGGTGGGGAAGACGACTACTGCTAGAATAGTATATGAAGCCATCCAAAGTGAGTTTGAAGTTTC
AY331206.1|Gossypium  barbadense ------GGGATGGGGGGGGTGGGGAAAACAACTCTTGCAAGGGTTGCTTACACTCAAATTTCACCTCATTTTGAAGGCAA
AF525135.1|Capsicum  annuum      ------GGGATGGGGGGG-TGGGGAAGACGACCCTAGCAAGAGTTATTTATGAGAATATTCAGAGTGAATTTGAAGGTGC
Clustal Consensus                                     *   *    **                 *        *

                                          90       100       110       120       130       140       150       160
                                 ....|....|....|....|....|....|....|....|....|....|....|....|....|....|....|....|

AF209496.1|Brassica_napus        TGACATTTTGATATGGATCGTG---GTGTCTCAAGGCGCAAATATTTCAAAGCTTCAAGAAGATATTGCACGGAAGCTAC
AF209499.1|Brassica_napus        TGACATTTTGATATGGATTGTG---GTGTCTCAAGGCGAAAATATTTCAAAGCTTCAAGAAGATATTGCACGGAAGCTAC
AF420476.1|Brassica_nigra        TG------TGATTTGGATTGTG---GTTTCTAAGGAGCTACATATAAGAAATATTCAAGAAGAGATCGCGAAGAAACTAG
AF433641.1|Brassica_oleracea     TGACATTGTGGTTTGGATGGTG---GTATCTAAGGATTTTCAGATCCAAAAGATTCAAGAAGAGATTGCGAAGAAGCTAA
AY746420.1|Citrus  trifoliate    TGATGTTGTGATCTGGGTGGTG---GTGTCTAAAGATTTACAGCTTGAAAAGATTCAAGAGAAAATTGGAAGAAGAATCG
AY130803.1|Citrus  maxima        TGATGTTGTGATCTGGGTGGTG---GTGTCTAAAGATTTACAGCTTGAAAAGATTCAAGAGAAAATTGGAAGAAGAATCG
AY746410.1|Citrus  trifoliate    TGATGTTGTCATTTGGGTGGTG---GTGTCTAAAGACATACAGCTTCAGAGAGTTCAAGAAAAAATCGGGGAAAGAATTG
FM992103.1|Gossypium  arboreum   CGATGTTGTAATTTGGGCACTG---GTGTCTAC-------GATGTTGGAAAGGTTCAAGATAGGATTGGTGGAAATCTTC
PS054                            CGATGTTGTAATTTGGGCACTG---GTGTCTACAAA----GATGTTGGAAAGGTTCAAGATAGGATTGGTGGAAATCTTC
AY331193.1|Gossypium  barbadense TGAAGTTGTTATCTGGGCATTG---GTGTCTAAAGAGTCCGATGTTGGAAAGATTCAAGATAGGATTGGTGGAAATCTTG
FJ769805.1|Gossypium_hirsutum    TGATGTTGTTATCTGGGCGTTG---GTGTCTAAAGATTACGATGTTGGAAAGATTCAAGATAGGATTGGTGTAAATCTTG
AK230460.1|Arabidopsis  thaliana TGATGTAGTGATATGGGTTGTG---GTATCTAAAGACTTTCAGTTGGAGGGCATTCAAGATCAGATTCTGGGGAGATTAC
PS157                            TAGAAAATTGATTTGGGTAACCAAAGTATCTCAAGTTTTCGATATTCGAAAGCTGCAAAAGGACATCGCTTCTGGGC---
PS016                            TAGAAAATTGATTTGGGTAACC---GTATCTCAAGTTTTCGATATTCGAAAGCTGCAAAAGGACATCGCTAAATCTC---
AF402768.1|Theobroma_cacao       TAAGAAATTGATTTGGGCAACT---GTATCCCAAGACTTTGATGTTCGACGGCTACAAAATGACATTGC---AAGCC---
AY747332.1|Arachis  hypogaea     TTATTTTCTTGCAAATGTAANGAAGACATGTGATAAAAATTGTATTGTTCAAGCACAAAAAGAACTTGTTGGCC--AT--
AY331206.1|Gossypium  barbadense AAGCTTTCTTGCTGATGTTCGAGAAGTTTCAAATAAATGGGGACTTGTTTCTTTACAGAAACAACTTCTTTCCCCAAT--
AF525135.1|Capsicum  annuum      ATGTTTTCTTCATGAAGTCAGAGACCGTTCATCAAAACACGGACTAGAGCGCTTGCAGGAGACACTTCTTTCTGAAAT--
Clustal Consensus                        *                   *               *          **   *     *

                                         170       180       190       200       210       220       230       240
                                 ....|....|....|....|....|....|....|....|....|....|....|....|....|....|....|....|

AF209496.1|Brassica_napus        ACCTTTGTGGCGAGGAATGGACAAACAAAAATGAAAGTGATAAAGCCGCAGAGATTCACACAGTTTTA---AAAAGGCAG
AF209499.1|Brassica_napus        ACCTTTGTGGCGAGGAATGGACAAACAAAAATGAAAGTGATAAAGCCGCAGAGATTCACACAGTTTTA---AAAAGGCAG
AF420476.1|Brassica_nigra        GATTGGATGGGGAAGACTGGAACCGGAAAGATAAAGAGCAAAAGGCGTGTGAGATACATAGTGTCTTG---AAGAGGAAG
AF433641.1|Brassica_oleracea     GCCTGACTGGCCAAGATTGGAACCAGAAAGATGAAGACCAAAAGTGCTGTGACATACATAATGTCCTA---AAGAGGAAG
AY746420.1|Citrus  trifoliate    GTTTTCTTGACGAATCATGGAAGAATGGAAGTCTTGAAGACAAAGCATCGGATATCTTGAGAATTTTG---AGCAAGAAG
AY130803.1|Citrus  maxima        GTTTTCTTGACGAATCATGGAAGAATGGAAGTCTTGAAGACAAAGCATCGGATATCTTGAGAATTTTG---AGCAAGAAG
AY746410.1|Citrus  trifoliate    GTTTCCTTGA------------GAATCAAAGTCTTGAAGGGAAAGCATCAGGTATCTTCAAGATCTTG---AGCAAGAAG
FM992103.1|Gossypium  arboreum   GGTTCTCAGACGACTCATGGAAGAATAAAAGTGTTGACCAGAAGGCTACAGATATCTATAGGGTGTTG---CGCTACAAG
PS054                            GGTTCTCAGACGACTCATGGAAGAATAAAAGTGTTGACCAGAAGGCTACAGATATCTATAGGGTGTTGAAACGCTACAAG
AY331193.1|Gossypium  barbadense GGTTTTCAGATGACTCATGGAAGAATAAAAGTGTTGACCGGAAAACTACAGATATCTATGGGGTGTTG---GGCGACAAG
FJ769805.1|Gossypium_hirsutum    GGTTTTCAGATGACTCGTGGAAGCATAAAAGTGTTGAAGAGAAAGCTGTAGATATCTATGGGGTCTTG---CGCAACAAG
AK230460.1|Arabidopsis  thaliana G---TCCTGACAAGGAATGGGAAAGAGAAACAGAAAGTAAGAAAGCCTCTCTCATAAACAATAACCTT---AAGAGAAAG
PS157                            AATTAGAGAGAAACTTGTCAGATGATGAAAGTACAATAGTCCGTGCAGGGAAGTTATCAAAAATGTTCAGAGGACAAATG
PS016                            AATTAGAGAGAAACTTGTCAGATGATGAAAGTACAATAGTCCGTGCAGGGAAGTTATCAAAAATGTTCAGAGGACAAATG
AF402768.1|Theobroma_cacao       AATTAGAAAAAACCTTGTCGGATGATAAAAATACAACTATTAGGGCTGGAGAGCTATTAGAAATGTTGAGGAAACAAGGG
AY747332.1|Arachis  hypogaea     -ATCAATGGAAGCTCGAGT-AATTTGATAAATGAGCATGATGGGAGGAGAATAATTCGGGCTTCTTTG---TGTCACAAA
AY331206.1|Gossypium barbadense  -CTTGCTTGACGGATGCTTCAATTTTTTCAATGTCTATGAAGGGAATGCTGTAATTAGCCACAAATTG---TCTCGTAAA
AF525135.1|Capsicum  annuum      -CCTTGTTGAAAAAGACCTAAGGATCAACAATTCATTTGAAGGGGCCAATATGCTAAAACAAAGACTA---CGGTACAAA
Clustal Consensus                                                                      *           *

                                         250       260       270       280       290       300       310       320
                                 ....|....|....|....|....|....|....|....|....|....|....|....|....|....|....|....|

AF209496.1|Brassica_napus        AGATTTGTGCTGATGCTAGATGACATATGGGAGAAAGTGGA---TTTAGAAGCCATCGGAGTTCCGGAACCA---ACCAT
AF209499.1|Brassica_napus        AGATTTGTGCTGATGCTAGATGACATATGGGAGAAAATGGA---TTTAGAAGCCATCAGAGTTCCGGAACCA---ACCAT
AF420476.1|Brassica_nigra        AAGTTTGTGTTGTTGTTGGATGACATATGGGAAAAAGTTAA---TCTAATGGAGATTGGAGTCCCTTACCCA---ACTAA
AF433641.1|Brassica_oleracea     AAGTTTGTTTTGCTGTTGGATGACATATTGGAAAAAGTTAA---TCTAGCGGAGATGGGAGTCCCGTATCCA---ACTGT
AY746420.1|Citrus  trifoliate    AAGTTTTTATTGTTATTGGATGATATTTGGGAGCGCGTTGA---TTTAACTAAAGTGGGTGTTCCTTTTCC---CAACCT
AY130803.1|Citrus  maxima        AAGTTTTTATTGTTATTGGATGATATTTGGGAGCGCGTTGA---TTTAACTAAAGTGGGTGTTCCTTTTCC---CAACCT
```

```
AY746410.1|Citrus  trifoliate   AAGTTTCTGCTATTGTTGGATGATATATGGGAAAGAATTGA---TTTAGCCAAAGTGGGTGTCCCTTTTCCAGCTAGTTC
FM992103.1|Gossypium  arboreum  AAGTTTGTTGTATTATTGGATGATTTATGGGAGAGGGTGGA---TTTGAACCAAGTTGGGATACCCAAACCA---AGCAA
PS054                           AAGTTTGTTGTATTATTGGATGATTTATGGGAGAGGGTGGA---TTTGAACCAAGTTGGGATACCCAAACCA---AGCAA
AY331193.1|Gossypium barbadense AAATTTGTTGTATTATTGACTGATTTATGGGAGAGGGTGGA---TTTGAACCAAGTTGGGATACCAAAACCA---AGCCA
FJ769805.1|Gossypium_hirsutum   AAATTTGTTGTATTATTGGATGATTTATGGGAGAGGGTGAA---TTTGAACCAAGTTGGGATACCAAAACCA---AGCCA
AK230460.1|Arabidopsis thaliana AAATTTGTGTTGTTGTTGGATGATCTCTGGAGCGAAGTAGA---TCTAATTAAGATAGGAGTTCCACCTCCA---AGTCG
PS157                           AGGTATGTGCTAATATTGGATGATGTATGGAGAAGCTTTAAATCCCTTGAGGATGTTGGAATCCTTGAGCCA---ACAAC
PS016                           AGGTATGTGCTAATATTGGATGATGTATGGAGAAGCTTT---TCCCTTGAGGATGTTGGAATCCTTGAGCCA---ACAAC
AF402768.1|Theobroma_cacao      AGCTTTTTGCTAATATTAGATGATGTATGGAGCAGCTTC---TCTTTTGAGGATGTTGGAATCCTTGAGCCA---ACAAC
AY747332.1|Arachis  hypogaea    AAGGTTCTTCTTGTTCTTGATGATATAAATGAAGAAAAACA---GTTGAAGAATTT---GGCCGAGGAGCAAGACTGGTC
AY331206.1|Gossypium barbadense AAGGTTCTTATTGTTCTTGATAATGTTGATAACATACAACA---CTTGAAATGCTT---GGTTGGAAGGCGTGATTGGTT
AF525135.1|Capsicum annuum      AAGATTCTTCTTTTTCTTGATGATGTTGATCACACGGATCA---GTTAAGATGCTTT--AGCCGGGGAGCATGAATGGTT
Clustal Consensus               *    * *  *  *  *   * *  *                    *        *             *

                                         330       340       350       360       370       380       390       400
                                ....|....|....|....|....|....|....|....|....|....|....|....|....|....|....|....|

AF209496.1|Brassica_napus       AGAAAATGGATGCAAAGTAGCATTCACCA---CCCGT-----------ACGTAAGAGGATGGGGGATCAT---CAACCCA
AF209499.1|Brassica_napus       AGAAAATGGATGCAAAGTAGTATTCACCA---CCCGTTCTGAGGATGTATGTAAGAGGATGGGGGATCAT---CAACCCA
AF420476.1|Brassica_nigra       AGAAAACAGATGCAAAGTAGTTTTCACCA---CTCGTTCTCTGGAAGTGTGTGGGCGCATGGGAGCTAAT---GTTGAGA
AF433641.1|Brassica_oleracea    AGAAAACGGTTGCAAAGTAATTTTCACCA---CTCGTTCTCTGGAATTGTGTGGGCGCATGGGAGCTGAT---GTTGAGA
AY746420.1|Citrus  trifoliate   AGAAAATAAATCCAAGATAGTCTTCACAA---CTCGTTTTCTTGAAATCTGTGGTGCAATGAAAGCTCAC---GAGTTCT
AY130803.1|Citrus  maxima       AGAAAATAAATCCAAGATAGTCTTCACAA---CTCGTTTTCTTGAAATCTGTGGTGCAATAAAAGCTCAC---GAGTTCT
AY746410.1|Citrus trifoliate    AAAAAATGCATCCAAGATAGTCTTTACAA---CTCGTCTTGAGAATGTTTGTGGCCTCATGGAAGCCCAA---AAGAAGT
FM992103.1|Gossypium  arboreum  AAGAAATGGTTCCAAACTTATTTTCACAA---CTAGGTCTTTGGCGGTATGTGGCGAAATGGAAGCTCGA---AAGAAAA
PS054                           AAGAAATGGTTCCAAACTTATTTTCACAAGGACTAGGTCTTTGGCGGTATGTGGCGAAATGGAAGCTCGAGCTAAGAAAA
AY331193.1|Gossypium barbadense AGAGAATGGTTCCAAACTTATTTTTACTA---CTAGGTCTTTGGAGGTATGTGGTGAAATGGAAGCTCAA---AAGAAAA
FJ769805.1|Gossypium_hirsutum   AGTAAATGGTTCCAAACTTATTTTCACTA---CACGGTCTTTGGAGGTATGTGGTGAAATGGGAGCTCGA---AAGAAAA
AK230460.1|Arabidopsis thaliana AGAAAATGGATCGAAGATAGTTTTCACCA---CACGTTCAAAGGAAGTTTGCAAGCACATGAAAGCTGAC---AAGCAGA
PS157                           AAATAATGGATGCAAATTAGTGTTGACCA---CACGTTCAGAAAGGGTTGTTCAAGGGTCAATGGGATTT---AAGAAAG
PS016                           AAATAATGGATGCAAATTAGTGTTGACCA---CACGTTCAGAAAGGGTTGTTCAATCAGTTATGGGATTT---AAGAAAG
AF402768.1|Theobroma_cacao      AGATAATGGATGCAAGTTAGTGTTGACAA---CACGCTCCGCAAAGGTTGTTCGAGAA---ATGGATTGT---AAGAAAG
AY747332.1|Arachis  hypogaea    TGGTTCTGGAAGCAGAATAATTATCACAA---CTAGAGACATGCATCTTCTAAAGATACATGATGCATAT---GAAATTT
AY331206.1|Gossypium barbadense CGGTTTAGGGAGTAGAATCATTGTAACAA---CAAGAGATGAGCATTTGTTGCGATCTTACCGAGTTGAT---GATATGT
AF525135.1|Capsicum annuum      TGGTCCCGGAAGCAGAGTGATTGTTACGA---CAAAAGACAAACACTTTCTTGTTAAGTACGAGGCTGAA---AGGATAT
Clustal Consensus                            *   *     * ** *   *                               *

                                         410       420       430       440       450       460       470       480
                                ....|....|....|....|....|....|....|....|....|....|....|....|....|....|....|....|

AF209496.1|Brassica_napus       TGCAAGTCAAGTGTTTGAAAGAAGATCAAGCATGAGAATTATTTAAACTCAAAATAGGA---TATGAAAAGTTA---AGA
AF209499.1|Brassica_napus       TGCAAGTCAAGTGTTTGAAAGAAGATCAAGCATGGGAATTATTTAAACTCAAAATAGGA---TATGAAAAGTTA---AGG
AF420476.1|Brassica_nigra       TTGCAGTCCAATGTTTGTCTCCTCATGATGCATTGGAGTTGTTCAAAAAGAAAGTTGGA---GAGATCACATTA---ACA
AF433641.1|Brassica_oleracea    TGGTAGTCCAGTGTTTGCCACCTCATGACGCATTGGAGTTGTTCAAAAAGAAAGTTGGA---GAGATCACGCTA---GGA
AY746420.1|Citrus  trifoliate   TAAAAGTAGAGTGTTTGGGACCTGAGGATGCATGGAGATTGTTTCGTGAGAATCTCCGA---AGAGATGTTCTT---GAC
AY130803.1|Citrus  maxima       TAAAAGTAGAGTGTTTGGGACCTGAGGATGCATGGAGATTGTTTCGTGAGAATCTCCGA---AGAGATGTTCTT---GAC
AY746410.1|Citrus  trifoliate   TCAAGGTGGAGTGTTTGGGAGACAACGAAGCTTGGGAATTGTTCCTACAGAAGGTCGGA---GAAGAGACTCTC---GGC
FM992103.1|Gossypium  arboreum  TTAAAGTGGAGTGCCTAAAATCAGAAGAGGCTTGGAAATTGTTTCAAGACAAGGTTGGA---GATGAAACCCTC---AAT
PS054                           TTAAAGTGGAGTGCCTAAAATCAGAAGAGGCTTGGAAATTGTTTCAAGACAAGGTTGGA---GATGAAACCCTCCGGAAT
AY331193.1|Gossypium barbadense TCAAAGTGGAGTGCCTGGAAACGGAAAAGGCTTGGGAATTGTTTCGATCCAAGGTTGGA---GATGAAACTCTC---AAC
FJ769805.1|Gossypium_hirsutum   TCAAAGTGGAGTGCCTGGAATCGGAGAAGGCTTGGGAATTGTTTCAAGACGAGGTTGGA---TATGAAACTCTC---AAC
AK230460.1|Arabidopsis  thaliana TAAAAGTTGACTGTTTGTCACCAGATGAAGCGTGGGAATTGTTTCGACTCACTGTTGGA---GATATCATATTA---AGG
PS157                           TTAAAGTTCCTTGTTTTTCTATGGAGGAAGCCATGAACTTATTCTTAAGCAAAGTTGGAAAACAAGACATGTTG------
PS016                           TTAAAGTTCCTTGTTTTTCTATGGAGGAAGCCATGAACTTATTCTTAAGCAAAGTTGGA---CAAGACATGTTG------
AF402768.1|Theobroma_cacao      TCCAAGTGCCCTATCTTTTAACGGATGAGGCGATGCAATTATTCTTAAGCAAAGTCGGA---CAAGACATGTTG------
AY747332.1|Arachis hypogaea     ACAATGTTGAAGTGTTAGGGGAAAGTGAATCCTTTGATCTCTTTCATTTGAAGGCTTTTAA--ACAACGAAA-G------
AY331206.1|Gossypium barbadense ACAAACCTACAACATTGAATCTTAAAGATGCACTTCGGCTTTTCAATTTGAAAGCTTTCGATGATGATACAATA------
AF525135.1|Capsicum  annuum     ACAGGATATGCTCTTTAAATTACGATGAAAGTTTACAACTGTTTAAACAACATGCTTTCAAGAAGAACCAC---------
Clustal Consensus                              *           *           * **

                                         490       500       510       520       530       540       550       560
                                ....|....|....|....|....|....|....|....|....|....|....|....|....|....|....|....|

AF209496.1|Brassica_napus       AGAGAACCCCGCATTGACGGCCTCGCTAGAAAGGTTGCGGAAAA---ATGTCATGGTTTCCCACTAGCGTTG--------
AF209499.1|Brassica_napus       AGAGAGCCCCGCATTGACGGCCTCACTAGAAAGGTTGCGGAAAA---ATGTCATGGGCTGCCACTAGCGTTGA-------
AF420476.1|Brassica_nigra       AGTCATCCAGAGATACCAGAGCTTGCGGCCATAGTTGCAAGAAA---ATGCCAAGGCCTCCCCCTCACCCTC--------
AF433641.1|Brassica_oleracea    AGTCATCCAAACATACCAGAGCTTGCTAGAATAGTTGCCAGAAA---ATGCCATGGCCTCCCCCTAGCCCTA--------
AY746420.1|Citrus  trifoliate   AACCATCCAGACATTCCTGAACTAGCCAGAAGTGTAGCTAAAGG---GTGTGCTGGCCTCCCCCTAGCCCTA--------
AY130803.1|Citrus  maxima       AACCATCCAGACATTCCTGAACTAGCCAGAAGTGTAGCTAAAGG---GTGTGCTGGCCTCCCCCTAGCCCT---------
AY746410.1|Citrus trifoliate    AGTCATCCTGATATTCCGGAATTAGCCAAAACCGTGGCAAAGGA---GTGTTGCGGATTACCCCTAGCGCTA--------
FM992103.1|Gossypium  arboreum  AGCCATCCAGATATTCGGGAACTTGCTAAACAAGTAGCCAAAAG---GTGCGGTGGCTTCCCCTTCGCTCTTAAG-----
PS054                           AGCCATCCAGATATTCGGGAACTTGCTAAACAAGTAGCCAAAAG---GTGCGGTGGCTTCCCCTTCGCTCTTAAGAAA--
AY331193.1|Gossypium barbadense AGCCATCCAGATATTCTAAATCTAGCTAAACAAGTAGCTGAAAG---GTGCGGTGGACTGCCGCTCGCCCTC--------
FJ769805.1|Gossypium_hirsutum   AGCCATCCAGATATCCCAAACCTAGCTAAACAAGTAGCTGAAAG---GTGCGGTGGCCTCCCCCTCGCCCT---------
AK230460.1|Arabidopsis thaliana AGCCATCAAGATATTCCCGCACTTGCAAGAATAGTTGCTGCAAA---ATGCCATGGCTTGCCACTTGCACTCAATGTCAT
PS157                           CCCAATCCAACTTTAGAATCATTAATGAAACTTGCTGTGAGGGAATTATATGATGGATTCCCCCTCGCCCTCAAGGTCCT
PS016                           CCCAATCCAACTTTAGAATCATTAATGAAACTTGCTGTGAGGAAAGAATATGATGGATTCCCCCTCGCCCTCAAGGTCCT
```

```
AF402768.1|Theobroma_cacao      CCCAATCCAAATTTAGAGTCGATTATGAAGGATGTTGTCTGCGA---ATGTGGTGGTCTCCCCCTCGCCCTCAAGGTCCT
AY747332.1|Arachis  hypogaea    CCTGCAGAAGAGTATTTGGATTTGTCCAAACAGGCGGTCAAATA---TTGTGCTGGATTCCCCCTCGCCCTCAAGGTCCT
AY331206.1|Gossypium barbadense CCAACAGAAGATTTTATTGAGCTTTCTAAACATATTGTACGTTA---TGCTGGTGGATTCCCCCTCGCCCTCAAGGTCCT
AF525135.1|Capsicum  annuum     CCCACCAAGGAATTTGCTGATCTGTCAGCTCAAGTTATAAAGCA---TACCGGTGGATTCCCCCTCGCCCTCAAGGTCCT
Clustal Consensus                                     *                              **  * **  *  *  *

                                       570       580       590       600
                                ....|....|....|....|....|....|....|....|...
AF209496.1|Brassica_napus       -------------------------------------------
AF209499.1|Brassica_napus       -------------------------------------------
AF420476.1|Brassica_nigra       -------------------------------------------
AF433641.1|Brassica_oleracea    -------------------------------------------
AY746420.1|Citrus_trifoliate    -------------------------------------------
AY130803.1|Citrus_maxima        -------------------------------------------
AY746410.1|Citrus_trifoliate    -------------------------------------------
FM992103.1|Gossypium_arboreum   -------------------------------------------
PS054                           -------------------------------------------
AY331193.1|Gossypium barbadense -------------------------------------------
FJ769805.1|Gossypium_hirsutum   -------------------------------------------
AK230460.1|Arabidopsis thaliana TGGCAAAGCCATGGTATGTAAAGAGACTGTACAAGAATGGCGT
PS157                           CGGCTAA------------------------------------
PS016                           CGGC---------------------------------------
AF402768.1|Theobroma_cacao      CGGC---------------------------------------
AY747332.1|Arachis hypogaea     CGGCA--------------------------------------
AY331206.1|Gossypium barbadense CGGC---------------------------------------
AF525135.1|Capsicum   annuum    CGGC---------------------------------------
Clustal Consensus
```
